# Endocrine Disruptors and Their Impact on Quality of Life: A Literature Review

**DOI:** 10.7759/cureus.83890

**Published:** 2025-05-11

**Authors:** Tane del Río Barrera, Kemberly Noemi Zambrano Ledesma, Maricarmen Aguilar Hernández, Karla Reyes Chávez, Alejandro Fabricio Aguirre Barajas, Dania Patricia Alvarez Vázquez, Gerardo Garcia Santiago, Alejandra Arias Castro

**Affiliations:** 1 General Medicine, Universidad Autónoma de Guadalajara, Guadalajara, MEX

**Keywords:** endocrine disruptors, enviromental exposure, hormonal imbalance, metabolic disorders, reproductive health

## Abstract

Endocrine-disrupting chemicals (EDCs) are widespread environmental contaminants that interfere with hormonal regulation, affecting metabolism, reproduction, neurodevelopment, and overall health. This review presents an overview of recent evidence on the health effects and biological mechanisms of EDCs, with a focus on their impact on hormonal balance and quality of life. An integrative literature review was conducted using 28 peer-reviewed articles published between 2020 and 2025, retrieved from databases such as PubMed, Scopus, and ScienceDirect. The selected studies explored the physiological and pathological effects of EDCs in humans. Compounds, such as bisphenol A, phthalates, and polychlorinated biphenyls (PCBs), can mimic or block hormones, disrupt endocrine signaling pathways, and bioaccumulate in tissues. Exposure, especially during critical developmental windows, is linked to metabolic disorders, infertility, neurodevelopmental delays, and hormone-sensitive cancers. Common exposure sources include food, air, household dust, water, and personal care products. Key mechanisms of action involve receptor binding interference, oxidative stress, and epigenetic alterations. EDCs pose a significant and growing threat to public health, warranting urgent regulatory measures, increased public awareness, and continued research to mitigate long-term health consequences.

## Introduction and background

In recent decades, there has been growing concern about the adverse health effects resulting from exposure to chemical substances known as endocrine disruptors. Within the complexity of human physiology, the endocrine system plays a major role in regulating hormones involved in development, growth, reproduction, and overall metabolism. However, this intricate system now faces a significant threat from silent invaders- endocrine-disrupting chemicals (EDCs). These are chemical substances with the alarming capacity to interfere with the endocrine system, which, as previously mentioned, is responsible for regulating various bodily functions, notably growth, development, metabolism, and reproduction. These disruptors can mimic or even block physiological hormones in our bodies, potentially causing serious negative health effects [1].

This process affects us from early development, especially during fetal life and the early years, where exposure to these compounds can be harmful even at minimal doses due to the sensitivity of these stages. During these periods, exposure may interfere with hormone synthesis, transport, metabolism, action, enzymatic processes, and oxidative stress. Some of the main compounds currently being studied include bisphenol A, perfluorinated compounds, and phthalates, which have been identified for their ability to bioaccumulate and cause long-term effects, even after brief exposure. These compounds are commonly found in daily life, such as in plastic bottles that act as synthetic estrogens, altering sexual development, the immune system, and metabolism. They are associated with early onsets of obesity, puberty, diabetes, cancer, and more. Despite scientific warnings issued decades ago, these compounds continue to be part of our daily environment and accumulate in the body, especially in adipose tissue, where they can remain for years, causing long-term individual and collective health effects. The combination of harmful compounds poses a risk beyond just physical impact, also affecting quality of life, productivity, healthcare costs, and emotional well-being [1].

Endocrine disruptors are present in our daily lives, prompting evaluation of their role in body physiology and hormonal balance. They impact development not only at a metabolic level but also neurologically and have been associated with the onset of certain tumors. This is because they can alter hormone levels by either inhibiting or stimulating their production, interfering with their metabolic pathways throughout the body, ultimately resulting in functional hormonal disruption. Several mechanisms are known to interrupt hormonal homeostasis, the most notable being: interaction with nuclear hormone receptors (estrogens, androgens, progesterone, thyroid, and retinoids) or via traditional non-steroidal pathways, transcriptional coactivators, and steroid biosynthesis enzymes [2].

## Review

An integrative literature review was conducted to analyze the effects of endocrine-disrupting chemicals (EDCs) on human health and quality of life. Scientific articles published between 2020 and 2025 were retrieved from indexed databases including PubMed, Scopus, and ScienceDirect. Search terms used included “endocrine disruptors,” “hormonal imbalance,” “quality of life,” “reproductive health,” “neurodevelopment,” “environmental exposure,” “toxicity prediction,” and “regulatory policy.”

Inclusion criteria consisted of original research articles, systematic reviews, meta-analyses, and regulatory reports published in peer-reviewed journals or official institutional sources, written in English or Spanish, and addressing the health effects, mechanisms of action, or regulatory perspectives of endocrine disruptors. Exclusion criteria included non-peer-reviewed articles, editorials, and studies not directly related to human health.

After initial screening, 31 sources were selected based on their relevance, scientific rigor, and contribution to understanding the relationship between EDC exposure and adverse health outcomes. The findings were summarized and organized thematically according to the systems affected (reproductive, metabolic, neurological, cardiovascular, etc.) and included emerging approaches such as omics technologies, AI-based toxicity prediction, and international regulatory efforts to provide a comprehensive overview.

Theoretical framework

Endocrine disruptors are considered substances that interfere with the body’s homeostasis, generating a significant impact on human health. Alterations have been observed in various systems, including reproductive, neurological, and metabolic systems, and in some cases, they are even considered tumor precursors. These effects are particularly evident in hormone-sensitive cancers such as ovarian, breast, and uterine cancer [2,3].

Despite growing public health concerns, many interactions and mechanisms between EDCs and diseases remain unclear. This review aims to better understand these relationships, inform detection methods, and support timely intervention strategies. It also underscores the role of exposure duration and identifies consumer products containing endocrine-disrupting compounds [2,3].

EDCs differ from hormone modulators. While modulators like soy or coffee can influence hormone levels without causing harm, disruptors interfere with hormonal function and lead to adverse health effects [4].

Several endocrine pathways are affected by EDCs. For instance, they influence thyroid metabolism via parathyroid hormone, impair adrenal hormone release (e.g., cortisol), and disrupt pancreatic insulin production, contributing to conditions like diabetes [4].

In recent years, a notable decline in fertility rates has been observed. This decline has been associated with the presence of endocrine disruptors, which play a major role in human reproduction. As previously mentioned, these disruptors are released in various forms such as pesticides, packaging, cosmetics, and more. They are linked to congenital anomalies and infertility, particularly due to exposure to polychlorinated biphenyls (PCBs) and dichlorodiphenyltrichloroethane (DDT), which possess toxic metabolites that persist in the environment and pose a risk to the population [5].

The primary pathways involved in sexual development are the androgenic and estrogenic pathways, both of which are affected by endocrine disruptors. One example is aneuploidy in germ cells, which can lead to miscarriage and infertility. Bisphenol A (BPA), for instance, has been shown to cause defects in meiosis and DNA damage, including strand breaks. Additionally, paternal exposure has been linked to epigenetic modifications such as hypomethylation of the H19 locus, histone acetylation, methylation, phosphorylation, and ubiquitination, as well as alterations in microRNA. Exposure to endocrine disruptors has also been associated with both female and male infertility, and with obesity, given that BPA, phthalates, and PCBs are lipophilic and tend to accumulate in adipose tissue.

It has also been shown that endocrine disruptors affect Leydig cells in the testes, located in the seminiferous tubules and responsible for spermatogenesis, promoting their apoptosis. Intracellular telomere shortening and oxidative stress have also been observed; for example, prolonged exposure to 1-nitropyrene (1-NP) can induce endoplasmic reticulum stress caused by reactive oxygen species (ROS), thereby affecting steroidogenesis. Additional abnormalities in the male reproductive tract include cryptorchidism, hypospadias, infertility, and testicular germ cell tumors [5-7].

Exposure to propylthiouracil has been shown to affect thyroid hormone levels when exposure occurs in utero or during lactation, significantly impacting neurological development and maternal behavior, with noticeable thyroid effects [8].

Endocrine disruptors present in dust interfere with the hormonal system, affecting key processes such as growth, development, reproduction, and metabolism. Dust serves as a reservoir for pollutants released from products and materials used in indoor environments [9].

The total burden of environmental exposures to chemical and physical contaminants includes fine particles, heavy metals, volatile organic compounds, and toxic gases. These substances can enter the body through respiratory, dermal, or digestive routes and may reach the central nervous system via the bloodstream or even the olfactory pathway. Pollution can activate mechanisms such as oxidative stress, neuroinflammation, and disruption of protective barriers like the blood-brain barrier, allowing toxins to enter the brain and contribute to neuronal deterioration [9].

Household dust and dust from other indoor spaces are a complex mixture of biological, chemical, and mineral particles. It may contain pesticide residues, phthalates, bisphenols, and heavy metals, originating from furniture, cleaning products, textiles, plastics, and other materials found in homes, offices, schools, and hospitals. For instance, homes may exhibit higher levels of phthalates and PBDEs due to the use of furniture and household items; offices often contain high levels of flame retardants from electronic equipment; and schools and daycare centers typically show elevated levels of phthalates and other compounds derived from toys and educational materials [9,10].

Two types of dust extracts were analyzed to better understand how these compounds can affect health. Total extracts include all chemicals present in the dust, offering a comprehensive view of potential exposure. Comparing these two types of extracts is crucial for identifying which substances are more likely to enter the human body and disrupt hormonal balance. These compounds have been shown to interfere with hormone receptors, leading to metabolic disorders, reproductive issues, and even neurodevelopmental abnormalities. This information is key to guiding public policies aimed at reducing exposure to these chemicals and preventing health risks [9].

On the other hand, epidemiological and pathophysiological evidence has demonstrated that chronic exposure to atmospheric pollutants, such as fine particles, nitrogen dioxide, and ozone, not only worsens existing cardiovascular diseases but also acts as an independent risk factor in their development. Among the most prominent mechanisms are oxidative stress, systemic inflammation, and endothelial dysfunction, all of which directly link air quality to cardiovascular system deterioration. Specifically, exposure to these pollutants is associated with a systemic inflammatory response reflected in elevated levels of pro-inflammatory cytokines like interleukin-6. This chronic inflammation accelerates atherosclerotic processes and increases the risk of cardiovascular events such as myocardial infarction and stroke [10,11].

It is important to emphasize that cognitive deterioration can also result from the pollutome-connectome axis. The pollutome refers to the total set of environmental pollutants, including airborne particles, heavy metals, pesticides, and endocrine disruptors, that can affect neurological health. Meanwhile, the connectome describes the brain’s structural and functional network, which, when disrupted, can lead to progressive cognitive deficits. This framework suggests that exposure to environmental pollutants influences the structure and function of the brain's connectome, accelerating neuronal aging and contributing to the development of neurodegenerative diseases such as Alzheimer’s and Parkinson’s [12].

In the context of respiratory diseases, nanotechnology has emerged as a promising strategy to improve drug delivery and minimize the adverse effects of potentially endocrine-disrupting compounds. Given that these disruptors are linked to chronic obstructive pulmonary disease (COPD), asthma, pneumonia, and conditions related to occupational exposures, nanomaterials may help mitigate their impact. These materials enhance the bioavailability and selectivity of medications, reducing systemic exposure to substances that could disrupt endocrine balance and optimizing the safety of therapies for patients with chronic lung diseases [13].

Certain chemical compounds commonly used in fragrances can act as endocrine disruptors with significant activity on the human hormonal system. These include phthalates, parabens, triclosan, and siloxanes, which are frequently found in personal care and household products. Exposure to these substances has been linked to various health problems, such as reproductive disorders, developmental abnormalities, and certain types of cancer [14].

Diabetes is an endocrine disease that affects a large number of people worldwide. Its pathophysiology involves several factors, among which environmental pollution stands out, particularly exposure to airborne pollutants such as PM2.5, ozone (O₃), nitrogen dioxide (NO₂), and other endocrine disruptors like plastics and pesticides. There is evidence linking chronic exposure to endocrine-disrupting chemicals with the development of type 2 diabetes, especially in pregnant women. This is mainly due to the chronic inflammation and insulin resistance these substances generate by affecting the pancreas and disrupting hormonal balance [15].

The presence of endocrine disruptors in food has a significant and often overlooked impact. Phytoestrogens, for example, are plant-derived compounds that can modulate and limit the action of human estrogens. Xenoestrogens, on the other hand, are synthetic substances found in pesticides, plastics, and industrial products that alter hormonal metabolism. To reduce exposure to these disruptors, it is recommended to choose organic foods, include a wide variety of foods in the diet, and avoid ultra-processed products [16].

One of the main routes of exposure to endocrine disruptors is through the skin via topical application. The absorption of chemical substances from cosmetics and personal hygiene products is possible, given the constant and prolonged exposure to these items. Such exposure has been associated with fertility issues, cancer, and neurological disorders. Nowadays, there is a notable reduction in the use of products containing lead and mercury. Nevertheless, it remains essential to adopt measures to minimize exposure to toxic substances, protect the environment and human health, and strengthen scientific evaluation processes to limit the presence of endocrine disruptors in personal care products.

Another route of exposure is ingestion. The interaction between endocrine disruptors and gut microbiota alters the microbial composition, promoting dysbiosis and disrupting metabolism, which can lead to diseases such as obesity and diabetes. Bisphenol exposure, for instance, has been linked to changes in the diversity and composition of gut microbiota. Therefore, identifying and avoiding these compounds or consuming them in smaller quantities is recommended [17,18].

Bone and joint pathologies rank third in incidence after cardiovascular and oncological diseases. New etiological factors and pathogenic mechanisms have begun to be investigated, and endocrine disruptors have been identified as contributors to the development of bone-related conditions. Substances such as diethylstilbestrol (DES), alkylphenols, bisphenol A (BPA), dioxins, and PCBs interfere with bone tissue development and regeneration by affecting the activity of osteoblasts and osteoclasts, altering calcium metabolism, and contributing to musculoskeletal disorders [19]. 

Exposure to endocrine disruptors (EDs) has been associated with various alterations in the genitourinary tract, including abnormalities in male genital development (e.g., hypospadias, cryptorchidism), erectile dysfunction, and fertility issues. In females, exposure can disrupt sexual development, leading to early menarche and increased risk of conditions like myometrial hyperplasia, endometriosis, and polycystic ovary syndrome (PCOS). Additionally, there is a heightened risk of cancers affecting reproductive organs. EDs can imitate, block, or alter the synthesis of natural hormones, particularly during critical developmental periods, and may induce epigenetic modifications in germ cells. These changes can lead to congenital malformations or increased cancer risk in offspring.

Endocrine disruptors also decrease the quality of both sperm and ova. Prenatal exposure to EDs has been linked to adverse effects on children's metabolic and neurological health. For example, previous studies have shown that exposure to mixtures of EDs during pregnancy can increase the risk of metabolic syndrome in childhood. Similarly, exposure to phthalates has been associated with reduced brain volumes and lower IQ scores in children [20-22].

Discussion

**Figure 1:**
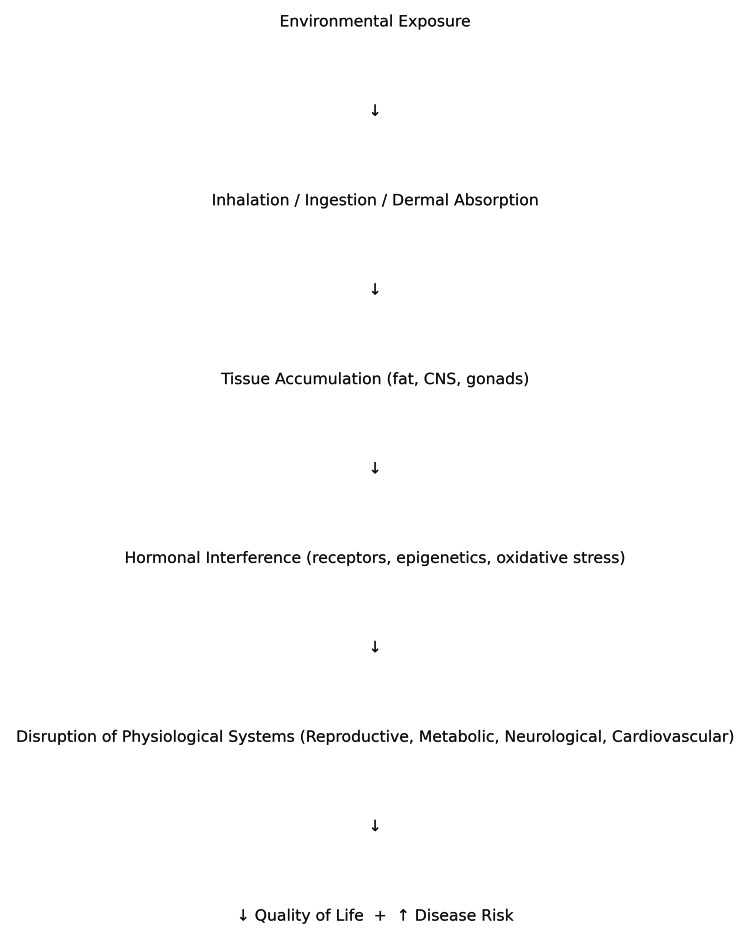
Pathway of endocrine disruptor exposure and its impact on human health This flowchart illustrates the general pathway by which endocrine-disrupting chemicals (EDCs) affect human health. It begins with environmental exposure through inhalation, ingestion, or dermal absorption. These substances accumulate in various tissues and interfere with hormonal signaling through mechanisms such as receptor binding, oxidative stress, and epigenetic alterations. As a result, multiple physiological systems—including reproductive, metabolic, neurological, and cardiovascular—are disrupted. This systemic interference ultimately leads to a decrease in quality of life and an increased risk of chronic diseases.

This review addresses the growing concern surrounding obesity and its relationship with endocrine-disrupting chemicals (EDCs), such as bisphenol A (BPA) and phthalates. These substances, present in a wide range of industrial and consumer products, can interfere with hormonal regulation and contribute to the development of obesity and metabolic disorders. Epidemiological studies have demonstrated that exposure to EDCs, especially during critical developmental periods, can alter adipogenesis and metabolic signaling. This suggests potential transgenerational effects that predispose individuals to obesity [2]. Figure 1 shows pathway of endocrine disruptor exposure and its impact on human health.

Recent advances in detection methods have identified harmful EDCs such as 17β-estradiol, perfluorooctane sulfonate (PFOS), and perfluorooctanoic acid (PFOA) using tools like enzyme-linked immunosorbent assay (ELISA) and fluorescent aptasensors. Notably, PFOS has shown greater toxicity to embryonic stem cells than PFOA, and its combined exposure with BPA exacerbates adverse cellular effects. These findings highlight the urgent need for more accessible and sensitive detection strategies [23].

Furthermore, EDCs have been shown to disrupt appetite regulation and modify gut microbiota composition, contributing to insulin resistance, systemic inflammation, and the onset of metabolic disorders such as obesity and type 2 diabetes [24]. 

In addition, BPA has also demonstrated neurotoxic effects in embryonic stem cell models, reinforcing the need for more stringent safety assessments and regulatory oversight of EDCs to mitigate their broader impacts on both human health and ecosystems [25].

Recent studies have emphasized the importance of global regulatory efforts in controlling exposure to endocrine disruptors. The European Union, through its Registration, Evaluation, Authorisation and Restriction of Chemicals (REACH) regulation, has introduced strict guidelines to phase out substances with endocrine-disrupting properties, while countries like Canada and Japan are also revising risk assessment frameworks for these chemicals. Despite such efforts, many regions, including Latin America and parts of Asia, still lack formal regulatory systems. This disparity highlights the need for international collaboration and harmonization of toxicological standards to protect vulnerable populations, especially children and pregnant women, who are more susceptible to the effects of EDCs [26-28].

Emerging technologies have opened new avenues for studying the mechanisms of action and long-term effects of EDCs. Omics-based approaches, such as metabolomics and transcriptomics, are being employed to detect subtle biochemical changes induced by low-dose chronic exposures. Moreover, artificial intelligence and machine learning models are increasingly used to predict EDC-related toxicity, offering faster and more comprehensive screening tools. These innovations, combined with the development of non-animal testing methods and in vitro models, are expected to improve the identification and regulation of potentially hazardous compounds in future research [29-31]. Table 1 shows summary of common endocrine disrupting chemicals, affected physiological systems, and reported health outcomes.

**Table 1:** Summary of common endocrine disrupting chemicals, affected physiological systems, and reported health outcomes BPA: Bisphenol A; PCBs: Polychlorinated biphenyls; DES: Diethylstilbestrol; NO₂: Nitrogen dioxide. This table categorizes frequently reported endocrine-disrupting compounds based on the physiological systems they affect and the associated adverse health effects. Reference citations supporting each outcome are included in the main text.

System Affected	Implicated Compounds	Reported Effects	References
Reproductive	BPA, phthalates, PCBs	Infertility, cryptorchidism, spontaneous abortion	[5-7, 20-22]
Neurological	BPA, phthalates, heavy metals	Cognitive delays, reduced brain volume	[9, 12, 20-21]
Metabolic	BPA, phthalates, pesticides	Obesity, insulin resistance, diabetes	[13, 15, 24]
Cardiovascular	Fine particles, NO₂, ozone	Systemic inflammation, endothelial dysfunction	[10-11]
Bone and Joint	DES, BPA, dioxins	Osteopenia, impaired bone regeneration	[19]

## Conclusions

This literature analysis highlights the impact of endocrine disruptors on our daily lives, supported by solid experimental and epidemiological evidence, emphasizing the damage they cause to human metabolism. Currently, they represent a threat to quality of life, as they silently affect the endocrine system, leading to adverse health effects in both humans and the environment. These substances are found in plastics, cosmetics, food, water, and pesticides. Although their impact is not immediate, constant and prolonged exposure leads to significant metabolic consequences.

It is necessary to implement population-wide interventions and stricter regulatory policies to reduce exposure and better assess the cumulative impact of these compounds. Therefore, awareness, education, and prevention must be considered key strategies to protect both current and future human quality of life. The existing data are sufficient to consider endocrine disruptors as a serious risk that must be addressed from both the standpoint of scientific research and public health.
